# Multivariate selection mediated by aridity predicts divergence of drought‐resistant traits along natural aridity gradients of an invasive weed

**DOI:** 10.1111/nph.18018

**Published:** 2022-02-28

**Authors:** Carter Carvalho, Rochelle Davis, Tim Connallon, Roslyn M. Gleadow, Joslin L. Moore, Akane Uesugi

**Affiliations:** ^1^ School of Biological Sciences Monash University Clayton Vic. 3800 Australia; ^2^ Biosciences and Food Technology Division School of Science RMIT University Bundoora Vic. 3083 Australia

**Keywords:** *Arctotheca calendula*, capeweed, clines, drought tolerance, invasive species, local adaptation, phenotypic selection, selection gradient

## Abstract

- Geographical variation in the environment underpins selection for local adaptation and evolutionary divergence among populations. Because many environmental conditions vary across species' ranges, identifying the specific environmental variables underlying local adaptation is profoundly challenging.
- We tested whether natural selection mediated by aridity predicts clinal divergence among invasive populations of capeweed (*Arctotheca calendula*) that established and spread across southern Australia during the last two centuries.
- Using common garden experiments with two environmental treatments (wet and dry) that mimic aridity conditions across capeweed’s invasive range, we estimated clinal divergence and effects of aridity on fitness and multivariate phenotypic selection in populations sampled along aridity gradients in Australia. We show that: (1) capeweed populations have relatively high fitness in aridity environments similar to their sampling locations; (2) the magnitude and direction of selection strongly differs between wet and dry treatments, with drought stress increasing the strength of selection; and (3) differences in directional selection between wet and dry treatments predict patterns of clinal divergence across the aridity gradient, particularly for traits affecting biomass, flowering phenology and putative antioxidant expression.
- Our results suggest that aridity‐mediated selection contributes to trait diversification among invasive capeweed populations, possibly facilitating the expansion of capeweed across southern Australia.

## Introduction

Species with broad geographical distributions often exhibit pronounced regional differentiation for fitness‐related traits, reflecting a history of selection for local adaptation (Endler, 1977, 1986; Kawecki & Ebert, 2004). Such intraspecific differentiation implies that the direction of selection changes rapidly with environmental conditions, which has broader implications for speciation (García‐Ramos & Kirkpatrick, 1997), the maintenance of genetic variation (Barton, 2001), the prevalence of fitness trade‐offs among environments (Hereford, 2009; Anderson *et al*., 2013) and the evolution of species’ ranges (Connallon & Sgrò, 2018; Polechová, 2018).

Environmental variation is a central feature of local adaptation, yet pinpointing the specific environmental variables generating selection for local adaptation is logistically challenging. Three traditional approaches – cline studies, field estimates of selection and reciprocal transplant experiments – are used widely in local adaptation research, yet all three are limited in what they can tell us about the specific environmental agents of selection that promote adaptive differentiation among populations (Wade & Kalisz, 1990; Wadgymar *et al*., 2017b). Cline studies, particularly those showing predictable and repeatable patterns of trait divergence (Weber & Schmid, 1998; Hoffmann & Weeks, 2007) or divergence in excess of neutral expectations (Leinonen *et al*., 2013; Hoban *et al*., 2016), are crucial for identifying traits and genes contributing to local adaptation. Field‐estimated associations between trait expression and fitness (i.e. phenotypic selection estimates; Kingsolver *et al*., 2001) potentially can validate whether contemporary selection aligns with geographical patterns of trait diversification, as expected under hypotheses of local adaptation (Colautti & Barrett, 2013). Reciprocal transplant experiments provide the clearest and most inclusive tests for local adaptation (Kawecki & Ebert, 2004), which predict that populations in their native habitats will outperform those transplanted from foreign environments (Hereford, 2009).

The combination of all three approaches can provide a fairly comprehensive picture of local adaptation: from its pervasiveness across a species’ range, to the traits and genes that facilitate it. A strength of these field‐based approaches is that they yield data reflecting the complexity of natural environments in which local adaptation has evolved, including fitness and selection estimates that are representative of the species’ natural environments, and clinal divergence estimates (i.e. from common garden experiments) representing evolved responses of source populations to ancestral environments. However, the environmental realism of such observational studies can be constraining when the goal is to identify specific environmental variables that determine the direction and strength of selection for local adaptation (Wadgymar *et al*., 2017b), as there is no way to conclusively evaluate which of the many ambient environmental variables that distinguish populations actually *caused* the geographical divergence for selection that underlies local adaptation (Wade & Kalisz, 1990; Caruso *et al*., 2017, 2020). Although manipulative experiments do not capture the richness and complexity of natural environments, the simplicity of these controlled experiments allows them to isolate effects of individual environmental variables on selection and thereby address questions that observational studies cannot (Wade & Kalisz, 1990; Caruso *et al*., 2017).

A recent meta‐analysis of > 200 studies and 7000 estimates of directional selection from globally distributed field populations (primarily terrestrial plants and animals) showed that climatic extremes associated with precipitation and evapotranspiration are correlated with temporal and spatial variation for directional selection (Siepielski *et al*., 2017), making water stress a compelling candidate environmental variable mediating selection and local adaptation. Although meta‐analyses of manipulative experiments confirm that abiotic factors profoundly affect directional selection (Caruso *et al*., 2017, 2020), few experiments have convincingly isolated effects of water stress from other environmental variables (Heschel *et al*., 2004; Volis *et al*., 2004; Heschel & Riginos, 2005; Sherrard & Maherali, 2006; Brachi *et al*., 2012; Ivy & Carr, 2012; Kenney *et al*., 2014; Lambrecht *et al*., 2017; Hamann *et al*., 2018; Metz *et al*., 2020). Even fewer have estimated both clinal divergence and selection from manipulative experiments to test whether water stress reliably predicts geographical divergence in nature (e.g. Heschel *et al*., 2004; Lambrecht *et al*., 2017; Hamann *et al*., 2018; Metz *et al*., 2020).

**Fig. 1:**
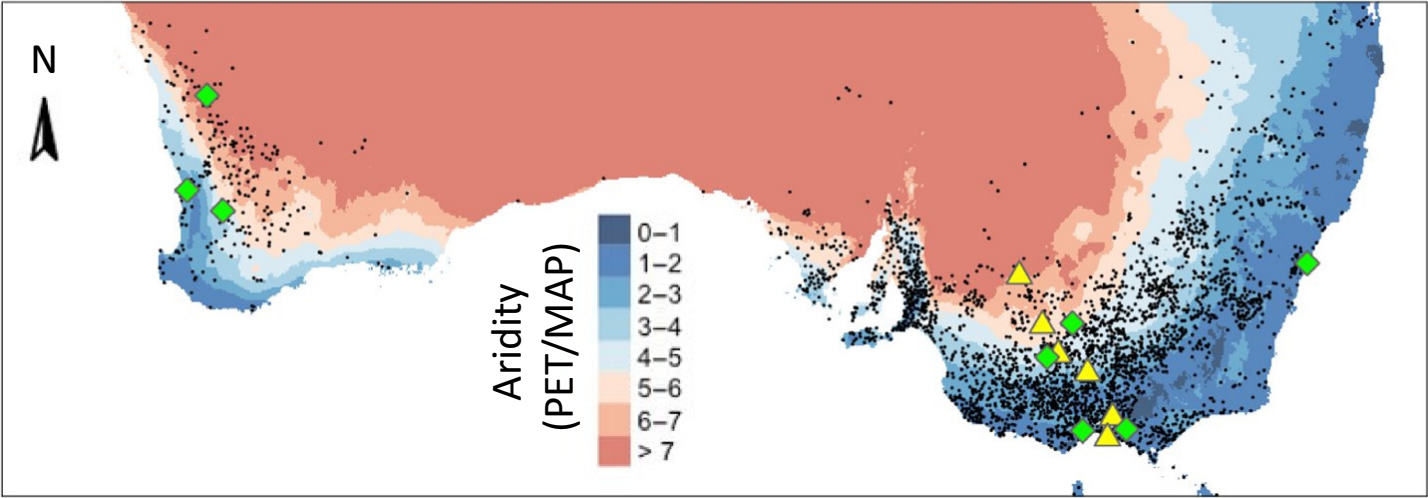
Natural aridity gradients in western and eastern Australia, the distribution of capeweed (*Arctotheca calendula*) observations in relation to these gradients (black dots), and the locations of populations sampled for the experiments. Source populations for the 2018 and 2019 experiments are marked in yellow and green, respectively.

Here, we test whether selection mediated by aridity predicts patterns of clinal divergence of the invasive annual capeweed, *Arctotheca calendula* (Asteraceae), across its distribution in Australia. Since its introduction from South Africa during the 19^th^ Century, capeweed’s range has expanded to encompass relatively wet southern coasts and dry interior regions within Australia (Atlas of Living Australia, 2010; Fig. 1), and local adaptation of drought‐resistance traits might have facilitated this rapid spread. To evaluate whether drought stress is likely to have driven divergent selection among invasive capeweed populations, we estimated fitness, selection and clinal divergence using capeweed populations that were sampled across natural aridity gradients in Australia (Fig. 1), and subsequently grown and phenotyped in common gardens with two water stress treatments: ‘wet’ and ‘dry’.

We tested four potential links between water stress, selection and evolutionary divergence across the aridity gradients. First, we tested whether capeweed populations were differentially adapted to aridity by comparing mean fitness of populations sampled across natural aridity gradients and grown in wet and dry common garden environments. Second, we estimated clinal divergence among populations for 13 plant traits that are thought to mediate drought escape, avoidance and tolerance strategies of adaptation in plants. Third, we tested whether drought conditions alter selection by contrasting directional selection for the same 13 traits in wet vs dry treatments. Finally, we evaluated whether multivariate selection in response to water stress predicts clinal divergence along natural aridity gradients by comparing multitrait vectors of differential selection between dry and wet environments with vectors of clinal divergence among populations.

We focus on traits spanning annual plant strategies of drought resistance (Ludlow, 1989; Kooyers, 2015). Plants may *escape* drought by growing rapidly and completing reproduction before dry conditions arise during annual cycles; in arid environments, selection may favour rapid growth rates, early flowering phenology, enhanced photosynthetic capacities and assimilation rates, and decreased vegetative biomass at maturity (Geber & Dawson, 1997; Kooyers *et al*., 2015). Plants may *avoid* dehydration during drought by evolving water‐conserving traits, including small, thick and succulent leaves, low stomatal conductance and increased root‐to‐shoot ratios that enhance water uptake (Ludlow, 1989; Dudley, 1996b; Sherrard & Maherali, 2006). Finally, annual plants may *tolerate* drought by producing secondary metabolites, such as phenolics and flavonoids, which potentially scavenge harmful reactive oxygen species (ROS) that accumulate under drought stress (Grace *et al*., 1998; Close & McArthur, 2002; Bettaieb *et al*., 2011; Agati *et al*., 2012). We tested whether selection predicts clinal divergence of 13 traits within and across all three strategies.

## Materials and Methods

### Study species

Capeweed (*Arctotheca calendula* (L.) Levyns) is an herbaceous annual plant native to South Africa, and invasive in Mediterranean climates globally (Global Biodiversity Information Facility). It was introduced into Australia in the 1830s (McIvor & Smith, 1973; Dunbabin & Cocks, 1999) and subsequently spread across regions substantially varying in rainfall (wet/cool southern coasts to dry/hot interior regions; Fig. 1). Capeweed germinates in autumn, forms deep taproots and basal rosettes during winter, and flowers in spring before onset of hot and dry summer conditions. Capeweed is self‐incompatible and insect‐pollinated.

### Common garden experiments

Two common garden experiments (2018 glasshouse; 2019 field) were conducted at Monash University (Clayton, Vic., Australia; lat. −37.9091°N, long. 145.1411°E).

#### Glasshouse experiment

In the 2018 experiment, conducted in a glasshouse, we investigated variation in drought resistance traits in six populations sampled along a transect spanning wet‐to‐dry capeweed range conditions in Victoria (Fig. 1; Supporting Information Table S1). In spring 2017, we bulk‐collected seeds from multiple plants within three to six patches in each of six populations (Fig. 1). Seeds were germinated, each patch separately, in potting mix placed in a growth chamber (12 h : 12 h, light : dark photoperiod, with 20°C day and 15°C night temperatures) in autumn 2018. After one month, we transplanted three to eight seedlings per patch (241 plants total; see Table S1) into 1‐l pots containing potting mix (Debco; Bella Vista, NSW, Australia) and Osmocote fertilizer (N : P : K = 19.4 : 1.6 : 5; Evergreen Garden Care Australia, Bella Vista, NSW, Australia). Plants were grown in the glasshouse under natural sunlight and temperature ranging from 7°C and 20°C during the experiment, similar to conditions during capeweed’s growing season. Individuals from each patch were split and assigned to two watering treatments designed to mimic mean winter rainfall conditions in the wettest and driest localities of sampled populations (i.e. Mornington vs Mildura, with mean monthly winter rainfall of 70.3 and 24.2 mm, respectively; Bureau of Meteorology, Australia). Wet treatment plants received 65 ml water twice weekly, whereas dry treatment plants received 45 ml once per week. Plants were placed randomly on three benches (‘blocks’) within the glasshouse. The experiment was terminated and biomass was harvested 15 wk after transplantation by which time most plants had senesced.

#### Field experiment

The 2019 experiment was conducted in an open field at Jock Marshall Reserve (Monash University, Clayton) to estimate selection in a semi‐natural environment of capeweed. Seeds for this experiment were collected from approximately 30 maternal plants each from eight populations distributed along transects in eastern and western Australia (Fig. 1; Table S1). We used seeds sourced from a broader range of capeweed’s Australian distribution to examine the predictability of population divergence across natural variation for aridity, and to increase trait variation and facilitate estimation of natural selection (Dudley, 1996a; Heschel & Riginos, 2005; Sherrard & Maherali, 2006). In July 2019, we germinated seeds in a growth chamber and transplanted one seedling per maternal family into a 1‐l pot (190 individuals, total, 7–36 individuals per population), placed randomly in the field plot (50 cm apart). Plants were grown in pots to allow estimation of root biomass at harvest. Plants were exposed to natural pollinators (predominantly European honeybees, which were abundant at the study site during the flowering period) and herbivores throughout the experimental period.

Individuals from each population were split equally into a wet treatment (*n* = 96), where plants were watered every other day unless it rained the day before, and a dry treatment (*n* = 95) reliant on ambient rainfall (mean monthly rainfall of 58.2 mm during the experiment). Although the experiment was conducted in a relatively wet part of capeweed’s invasive range, the dry treatment clearly reduced water availability to plants. Although we did not measure soil moisture in each pot, we suspect that the dry treatment in the field experiment was less severe than the glasshouse dry treatment, judging from the smaller difference in plant biomass between the water treatments in the field (see Table 1). We terminated the treatment and harvested plants after 15 wk (November) when most plants had senesced.

**Table 1:** Results of univariate analyses testing for the effects of capeweed’s source population aridity of (‘aridity’) and water treatment on plant phenotypes (standardized with mean = 0, SD = 1) in 2018 glasshouse and 2019 field experiments.

	2018 Glasshouse		2019 Field	
	Aridity	Treatment	Aridity	Treatment
*Escape traits*				
Growth rate	**0.011 (9.0)**	**−0.28 (370.1)**	0.003 (0.3)	**−0.18 (64.6)**
*F*_v_/*F*_m_	0.0001 (0.3)	0.001 (0.5)	0.0001 (0.2)	0.005 (1.9)
Photosynthesis	**0.44 (15.6)**	**−3.8 (76.4)**	0.030 (0.0)	**−5.88 (52.8)**
Biomass	**−0.041 (14.8)**	**−1.5 (1251.3)**	**−0.062 (18.7)**	**−0.96 (235.3)**
Days to flowering	−0.005 (0.0)	**0.78 (9.9)**	**−0.047 (14.5)**	−0.035 (0.4)
*Avoidance traits*				
Root‐to‐shoot ratio	−0.001 (0.0)	**0.30 (69.7)**	−0.04 (2.8)	**0.53 (25.7)**
Leaf size	−0.034 (4.2)	**−0.47 (51.2)**	0.009 (0.4)	**−0.38 (34.8)**
Succulence	0.065 (1.8)	**0.46 (5.9)**	0.042 (0.7)	**−2.53 (126.3)**
Specific leaf area	−0.016 (0.29)	0.41 (5.2)	0.038 (5.8)	−0.045 (0.4)
Stomatal conductance	**0.012 (13.9)**	**−0.093 (58.4)**	0.006 (0.7)	**−0.17 (26.9)**
*Tolerance traits*				
Chlorogenic acid	0.016 (0.5)	**0.35 (15.0)**	0.019 (1.8)	−0.038 (0.4)
Unknown phenolic acid	0.35 (4.4)	**−2.29 (12.6)**	**0.28 (28.9)**	**−0.74 (10.8)**
Unknown flavonoid	**0.12 (21.1)**	**0.39 (15.0)**	0.072 (3.7)	−0.290 (3.2)

Estimates of cline and drought treatment, along with *F*‐values (in parentheses) are presented. Estimates in bold indicate significance (*α* = 0.05) after Holms–Bonferroni adjustment.

In both experiments, we used field‐collected seeds rather than seeds generated by crosses in a common glasshouse environment. Although our approach does not control for maternal effects, we adopted it for two reasons. First, the small sizes of capeweed seeds suggest that maternal effects on plant phenotype at later developmental stages might be small (we return to this point in ‘the Discussion section’). Second, artificial crosses in a glasshouse also are potentially subject to bottlenecks and selection that introduce additional biases (Colautti & Barrett, 2013).

### Plant trait measurements

We measured 13 traits that are thought to mediate drought resistance in annual plants (Ludlow, 1989). Drought escape traits included growth rate, maximum photosynthetic capacity (*F*_v_/*F*_m_), photosynthetic assimilation rate, final biomass and days to first flower (Ludlow, 1989; Kooyers, 2015). Root‐to‐shoot ratio, leaf size, leaf succulence, specific leaf area (SLA) and stomatal conductance are linked to plants’ dehydration avoidance strategy (Ludlow, 1989; Kooyers, 2015). We quantified three leaf phenolic compounds that potentially are associated with plants’ drought tolerance strategy (as antioxidants; Grace *et al*., 1998; Close & McArthur, 2002). Many phenolic compounds, including phenolic acids and flavonoids are known to effectively scavenge reactive oxygen species (ROS; Grace *et al*., 1998; Close & McArthur, 2002; Agati *et al*., 2012), and can be induced in response to abiotic stresses, such as low temperature, high radiation (Grace *et al*., 1998) and drought (Bettaieb *et al*., 2011). We carried out an initial scan of phenolic acids and flavonoids in capeweed, using the approach of Keinänen *et al*. (2001), and identified three major compounds: chlorogenic acid, an unknown phenolic acid and an unknown flavonoid. We chose these compounds for the current analysis based on documented antioxidant properties of chlorogenic acid (Grace *et al*., 1998) and the high concentrations of all three compounds in capeweed leaves, implying important ecological functions.

#### Growth and phenology

Growth rate was estimated by counting rosette leaves, 0 and 8 wk after transplanting. Flowering was monitored every 2–3 d, beginning Week 9, to determine flowering time. At Week 15, above‐ and belowground biomass was harvested, oven‐dried at 60°C for 72 h, and weighed. Total biomass and root‐to‐shoot ratio were calculated.

#### Photosynthetic physiology

The potential maximum yield of photosystem II (dark‐adapted chlorophyll fluorescence, *F*_v_/*F*_m_, a metric of potential photosynthetic efficiency of light reactions) was measured during Week 4 on the most recently fully expanded leaf, per plant, using a PAM‐210 Chlorophyll Fluorometer (Waltz, Effeltrich, Germany). We took measurements after dark (22:00–04:00 h) to ensure acclimatization to dark.

Photosynthetic assimilation rate and stomatal conductance were measured over 3 d in Week 5 on the most recently expanded leaf, using a portable photosynthesis system (Li‐6400; Li‐Cor Biosciences, Lincoln, NE, USA). Measurement conditions were: 400 ppm CO_2_ concentration, 30–50% humidity, 23°C block temperature, 500 μml flow rate and photosynthetically active radiation (PAR) at 1000 μmol m^−2^ s^−1^ (PAR intensity yielding maximum photosynthetic rate in preliminary tests). For each plant, we took the average of three measurements for each trait.

#### Leaf morphology

In Week 7, we collected, scanned and determined leaf area of the largest fresh leaf from each plant, using ImageJ (v.1.51). We obtained both FW and DW, the latter after drying 48 h at 45°C. Leaf succulence was calculated as ((fresh weight–dry mass)/dry weight) (Ogburn & Edwards, 2012) and SLA as (leaf area/dry weight).

#### Leaf secondary metabolites

We collected *c*. 200 mg of a most recently expanded leaf in Week 8, flash‐frozen in liquid nitrogen, then freeze‐dried. Powdered sample (50 mg DW) was extracted in 1 ml 80% methanol, centrifuged for 20 min and filtered through a 0.45‐μm pore membrane (Pall Corp., Cheltenham, Vic., Australia). We analyzed the sample using high‐performance liquid chromatography (HPLC, Infinity 1260; Agilent Technologies, Australia, Mulgrave, Vic., Australia), equipped with C18 reserve‐phase column (Poroshell 120 EC‐C18, 2.7 μm, 150 mm × 3.0 mm; Agilent Technologies). The elution method was: 0–2 min, 0%–12% of B; 2–3.3 min, 12%‐18% of B, and 3.3–10 min, 18%–58% of B, with a flow rate of 0.5 ml min^−1^ and injection volume of 5 µl (Keinänen *et al*., 2001; van Boheemen *et al*., 2019b). We found three phenolic compounds at consistently high concentrations, including chlorogenic acid (Retention time (RT) = 9.6 min; quantified at 320 nm using a standard curve for chlorogenic acid: Sigma‐Aldrich), an unknown phenolic acid (RT = 12.50 min; quantified at 320 nm) and an unknown flavonoid (RT = 12.50 min; quantified at 360 nm).

#### Fitness

We used fecundity (seed production per individual; 2019 field experiment) as a fitness measure. Because capeweed is an annual plant, seed count is a good approximation of lifetime fitness (Dudley, 1996a; Colautti & Barrett, 2013). To estimate total seeds per plant, we bagged three to five capitula per plant following natural pollination in the field. Bagged capitula were used to estimate seed number per g. Remaining capitula were collected as seeds matured, dried and weighed to obtain the total number of seeds per plant. Relative fitness was calculated by dividing by mean seed production of the treatment.

Although measurements were taken at different time points in the life cycle for different traits (for logistical reasons), each trait was measured at approximately the same growth stage per experiment, allowing for comparisons between experiments.

### Statistical analyses

Analyses were performed using R (v.3.6.0) and RStudio (v.1.2.1335).

#### Aridity

Mean annual precipitation data were extracted from the Worldclim Bioclimate Dataset at 30s (http://worldclim.org). Potential evapotranspiration (PET) data were obtained from CGIAR‐CSI (http://www.cgiar‐csi.org). Local aridity per source population (hereafter ‘source location aridity’) was calculated as [PET/mean annual precipitation], in which larger values (intuitively) correspond to greater aridity (Fig. 1; note that our aridity metric is the inverse of the Aridity Index defined by Middleton & Thomas, 1992).

#### Clinal divergence and plasticity

In order to test whether source location aridity and watering treatment influenced plant phenotypes, we conducted a univariate analysis of the relation between local aridity and trait expression, separately for each experiment. Trait values were log‐ or square‐root‐transformed, when necessary, before analysis. For the 2018 experiment, population averages (least square means, LSMEANS) were calculated using a linear mixed model (*lmer* function) with population × treatment as fixed effects, and patch within a population and experimental block as random effects. LSMEANS for the 2019 field experiment were calculated with a linear model (*lm* function) with population × treatment as predictors. We then performed linear regression analyses with aridity × treatment as predictor variables and LSMEANS (calculated above) as dependent variables (following Wadgymar *et al*., 2017a; Lasne *et al*., 2018). We corrected α values for multiple testing with Holms–Bonferroni adjustments. *Post hoc* analyses (*emtrends* function) tested for significant (i.e. nonzero) cline slopes in each treatment (Table S2).

In order to evaluate multivariate patterns of clinal divergence between experiments, we calculated the angle (*θ*) between clinal divergence vectors, Δ ***z*_ghouse_** and Δ ***z*_field_** (each including 13 regression slopes of trait expression on source location aridity):

$$\theta =\cos^{‐1}\left(\frac{\sum_{i}^{n}\Delta z_{\text{ghouse},i}\Delta z_{\text{field},i}}{∥\Delta z_{\mathrm{ghouse}}∥\cdot ∥\Delta z_{\mathrm{field}}∥}\right)$$

($\Delta z_{\text{ghouse},i}$ and $\Delta z_{\text{field},i}$, slopes of the *i*th trait in the glasshouse and field experiments, respectively; *n*, number of traits; and $∥\Delta z_{\mathrm{ghouse}}∥=\sqrt{\left(\sum_{i}^{n}\Delta z_{\text{ghouse},i}^{2}\right)}$ and $∥\Delta z_{\mathrm{field}}∥=\sqrt{\left(\sum_{i}^{n}\Delta z_{\text{field},i}^{2}\right)}$, the magnitudes (norms) of divergence; an acute angle between vectors (less than orthogonal) indicates similarity in the overall direction of divergence.)

In order to assess uncertainty around *θ*, $∥\Delta z_{\mathrm{ghouse}}∥$ and $∥\Delta z_{\mathrm{field}}∥$ estimates, we used a Bayesian framework (mcmcpack package) to estimate the cline slope for each trait while controlling for treatment effects. We first standardized values of the 13 traits within each treatment and experiment (i.e. mean = 0, variance = 1) to facilitate comparison of clinal patterns across traits and treatment groups. Standardized values were combined within each experiment to obtain population‐level LSMEANS. We obtained 10 000 samples of the marginal posterior distribution of LSMEANS for each trait using mcmcglmm with population as a fixed factor and treatment (and patch and block for the glasshouse experiment) as random factors. Each MCMC sample of LSMEANS then was used to estimate the cline slope, $\Delta z_{\text{ghouse},i}$ and $\Delta z_{\text{field},i}$, using linear regression with aridity as a predictor. These results were used to generate 10 000 vectors of $\Delta z_{\mathrm{ghouse}}$ and $\Delta z_{\mathrm{field}}$, along with their norms and the angle between them, from which mean estimates and 95% credible intervals (CIs) of the parameters were calculated. Similar analyses were conducted within each experiment, but between treatments, to test how water treatments affect clinal patterns (see Fig. S2, see later).

#### Fitness along aridity gradients

We examined the relationship between source location aridity and fitness in wet and dry treatments of the 2019 field experiment by calculating relative fitness of individuals in each treatment, using a linear model to estimate population‐level LSMEANS. We then conducted a linear regression analysis on combined LSMEANS from both treatments, with aridity × treatment as predictors. *Post hoc* analysis with the *emtrends* function evaluated statistical significance of clines per treatment.

#### Phenotypic selection gradients

We estimated selection gradients for each trait and treatment using the 2019 field experiment data. To measure selection, we treated each individual as an independent sample. Selection gradients ($\beta_{i}$ for trait *i*) are estimates of the linear association between trait expression and relative fitness while controlling for indirect effects of selection on correlated traits (Lande & Arnold, 1983). For each treatment, we conducted an ordinary least squares (OLS) multiple linear regression to estimate *β* for each trait, with relative fitness as the dependent variable and standardized values of the 13 traits (mean = 0 and stdev = 1; 2019 field experiment) as independent variables. Our modest sample sizes precluded estimation of nonlinear (quadratic and correlational) selection.

All individuals survived to the flowering stage and, thus, all traits were measured for each plant. However, because many plants in the dry treatment (27 of 95) did not produce mature seeds (zero‐inflation), we evaluated significance of selection gradients using an aster model (*aster* function; Shaw *et al*., 2008; Geyer & Shaw, 2010), which combines binary data (i.e. reproduced or not) and count data (i.e. number of seeds in individuals that reproduced) into a single model. Statistical significance for the wet treatment was evaluated using a generalized linear model with negative binomial function (*glm.nb*). The aster model was not applied to wet treatment data because all individuals produced at least some seeds, which violates the model’s assumptions (Shaw *et al*., 2008).

#### Differences in selection between wet and dry treatments

We estimated differences in selection between treatments by calculating the angle between selection gradient vectors for the 13 traits in wet and dry treatments, ***β*_wet_** and ***β*_dry_**, respectively, and the magnitudes of directional selection per treatment (***β*_wet_** and ***β*_dry_**). Uncertainty was assessed using the Bayesian framework (*MCMCregress* function) outlined above (see Gosden *et al*., 2012, for a similar analysis).

#### Alignment between clinal divergence and differences in selection between water treatments

Intuition suggests that *if* aridity is an important driver of local adaptation in natural capeweed populations, then directional selection estimates from environments that differ solely in aridity (e.g. wet and dry common garden treatments) should be predictive of patterns of phenotypic divergence among populations sampled across natural aridity gradients (Fig. S1). To formally explore this verbal prediction, we developed an idealized model of: (1) adaptive clinal divergence for a set of *n* genetically independent quantitative traits; and (2) linear selection gradients for the same traits in a common garden experiment with two environmental treatments. The model justifies comparisons between vectors of trait divergence among populations and selection differences between experimental treatments (we estimate both) as a means of quantifying effects of spatial variation in aridity on selection for local adaptation. Assuming that each trait is able to evolutionarily track its local optimum, and if divergence is driven by selection mediated by aridity, then the vector of clinal divergence for the set of traits will align with the vector of selection gradient differences between dry and wet environments (see Fig. S1; Notes S1). Heterogeneity among traits in the strength of stabilizing selection generates a degree of mismatch between vectors, even when aridity is the sole environmental variable affecting local selection.

Our empirical estimates of clinal divergence (vector Δ ***z***) include the set of regression slopes of trait expression on source location aridity, using the Bayesian framework (mcmcglmm), as above, with data from both treatments and experiments (Table S3). The differential selection vector (Δ ***β*** = ***β*_dry_** – ***β*_wet_**; $\Delta \beta_{i}=\beta_{\text{dry},i}‐\beta_{\text{wet},i}$ for trait *i*) is based on 10 000 MCMC posterior samples described above. MCMC estimates of Δ ***z*** and Δ ***β*** were used to calculate the angle between vectors and 95% CIs.

## Results

### Plasticity and clinal differentiation

Our common garden experiments suggest that capeweed phenotypes are shaped by both genetic and environmental factors. Initial, univariate analyses revealed no significant aridity‐by‐treatment interactions, suggesting that the relationship between traits and aridity is similar in the two water treatments. Removing the interaction term, we found that plant phenotypes differed between dry and wet treatments in 11 of 13 traits in the glasshouse experiment and eight traits in the field experiment (Table 1). Dry conditions consistently decreased growth rate, photosynthesis, final biomass, leaf size, stomatal conductance and phenolic acid concentration, and increased root‐to‐shoot ratio. Several traits showed significant clinal divergence across the aridity gradient (four and three traits, respectively, in glasshouse and field experiments), including consistently negative correlations between biomass and source population aridity (Table 1; Fig. S2).

Estimates of multivariate clinal divergence were similar between the glasshouse and field experiments, with the angle between vectors of divergence in each experiment significantly less than orthogonal (estimated angle: *θ* = 53.8°, CI = (40.3°, 67.1°)). The magnitudes of multivariate clines were > 0 in both experiments, and similar between experiments (estimated norms: $∥\Delta z_{\mathrm{ghouse}}∥$ = 0.38, CI = (0.31, 0.45); $∥\Delta z_{\mathrm{field}}∥$ = 0.36, CI = (0.30, 0.41)). Clinal divergence was similar between water treatments in the glasshouse ($\theta =56.4^{\circ }$, CI = (38.8°, 76.1°)) and field experiments ($\theta =48.5^{\circ }$, CI = (32.2°, 75.4°)).

Combining data across experiments (using the Bayesian approach outlined in ‘the Materials and Methods section’), we found that populations sampled from arid regions had enhanced drought escape ability, via faster growth, higher photosynthetic rate and earlier flowering, and were smaller than populations from wetter localities (Table S3; Fig. 4). Surprisingly, arid populations expressed traits that *reduced* their ability to avoid dehydration, including higher SLA, and greater stomatal conductance. Finally, arid populations exhibited increased leaf concentrations of all phenolic compounds.

### Adaptation to local aridity environments

**Fig. 2:**
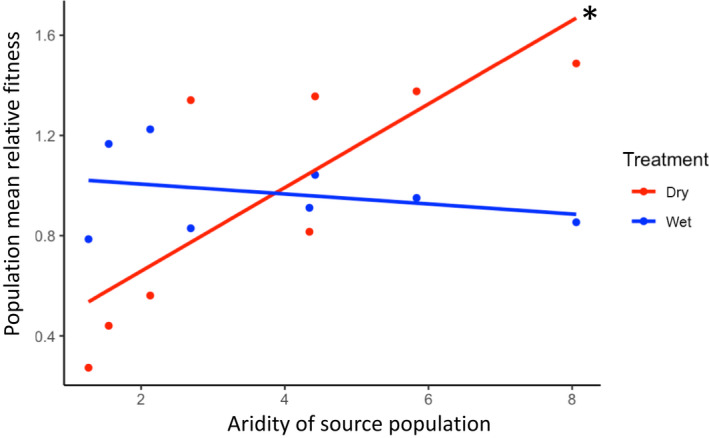
Relationship between relative fitness (seed set/mean seed set) of capeweed (*Arctotheca calendula*) and local aridity conditions (source location aridity: [PET/mean annual precipitation]) for eight experimental populations (for 2019 experiment) sampled across aridity gradients in eastern and western Australia (see Fig. 1), and grown in dry (red) and wet (blue) common garden environmental treatments. The asterisk indicates that the slope of regression in the dry treatment is significantly different from zero (no overlap in 95% confidence intervals estimated with the *emtrends* function).

If capeweed populations are locally adapted with respect to conditions of aridity, we expect populations sourced from relatively arid sites to perform better in dry treatments, and those from wetter sites to perform better in wet treatments. We tested this hypothesis using fitness (seed set) data from the 2019 field experiment. We identified a significant effect of treatment (wet vs dry) on the relationship between source location aridity and relative fitness of experimental populations (aridity‐by‐treatment interaction; *F*_1,12_ = 10.5, *P = *0.007). A *post hoc* test found a positive relationship between relative fitness and source location aridity in the dry treatment (slope = 0.17, CI = (0.07, 0.26)) and a negative, although nonsignificant, point estimate in the wet treatment (slope = −0.02, CI = (−0.1, 0.07); Fig. 2).

### Divergent selection between wet and dry environments

**Fig. 3:**
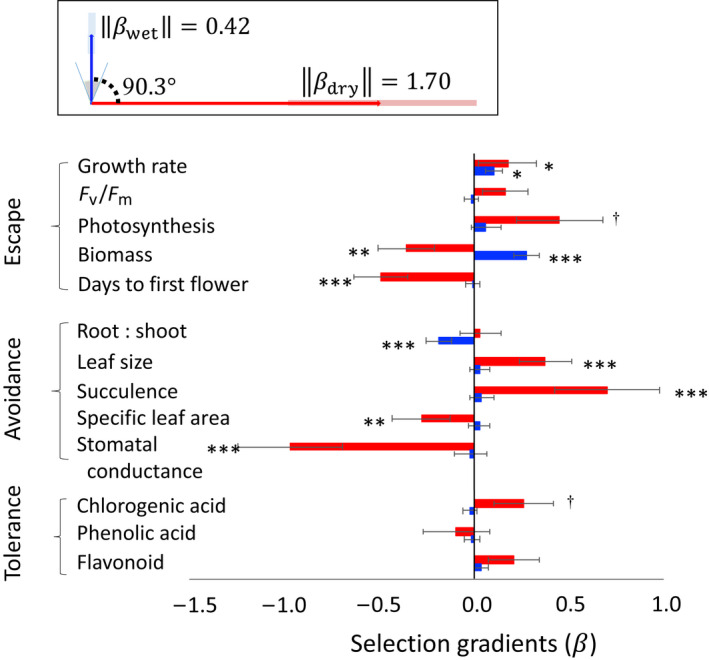
Estimated linear selection (***β***) on focal traits of capeweed in dry (red) and wet (blue) environmental treatments. Asterisks indicate statistical significance (†, *P* < 0.1; *, *P* < 0.05; **, *P* < 0.01; and ***, *P* < 0.0001) in the aster model (dry treatment) or negative binomial model (wet treatment); SEs are shown. The top panel shows vectors of total selection in dry and wet treatments, with the angle between vectors representing their alignment in 13‐dimensional trait space. The grey region represents the 95% credible interval (CI) for the estimate of the angle using MCMC sampling. The length of each arrow ($∥\beta_{\text{wet}}∥$ and $∥\beta_{\text{dry}}∥$) represents the magnitude of total selection (a selection gradient vector’s Euclidean norm), with shaded regions indicating 95% CI in the estimates.

The direction and strength of multivariate directional selection differed substantially between dry and wet treatments. Wet and dry selection gradient vectors had orthogonal orientations (*θ* = 90.3°, CI = (67.8°, 111.3°)), with stronger selection in the dry treatment ($∥\beta_{\mathrm{dry}}∥$ = 1.70, CI = (1.15, 2.29); $∥\beta_{\mathrm{wet}}∥$ = 0.42, CI = (0.31, 0.54); note the nonoverlapping CI; Fig. 3).

Seven of 13 selection gradient estimates differed significantly (and two marginally) from 0 in the dry treatment, whereas three of 13 were significant in the wet treatment (Table 2; Fig. 3). In the dry treatment, selection favoured individuals that grew faster, flowered earlier, had lower final biomass, had larger, thicker and more succulent leaves, and lower stomatal conductance (Fig. 3; red bars). In the wet treatment, selection favoured faster growth and larger biomass, with less biomass allocated towards roots (Fig. 3, blue bars).

**Table 2:** Linear directional selection (*β* (±SE)) on focal traits of capeweed, estimated in dry and wet treatments.

	Dry treatment		Wet treatment	
	*β* (±SE)	*z*‐value (*P*)	*β* (±SE)	*z*‐value (*P*)
*Escape traits*				
Growth rate	**0.17 (±0.15)**	**2.15 (0.032)**	**0.10 (±0.05)**	**2.51 (0.012)**
*F*_v_/*F*_m_	0.16 (±0.12)	1.40 (0.16)	−0.02 (±0.04)	−1.08 (0.28)
Photosynthesis	0.45 (±0.23)	1.85 (0.06)	0.06 (±0.08)	0.77 (0.44)
Biomass	**−0.36 (±0.15)**	**−2.32 (0.02)**	**0.28 (±0.07)**	**5.48 (< 0.0001)**
Days to first flower	**−0.49 (±0.14)**	**−3.75 (0.0002)**	−0.01 (±0.04)	−0.64 (0.51)
*Avoidance traits*				
Root‐to‐shoot ratio	0.03 (±0.11)	0.84 (0.40)	**−0.19 (±0.07)**	**−3.48 (0.0005)**
Leaf size	**0.37 (±0.13)**	**3.19 (0.001)**	0.03 (±0.05)	0.66 (0.51)
succulence	**0.70 (±0.27)**	**3.39 (0.001)**	0.04 (±0.06)	0.81 (0.42)
Specific leaf area	**−0.28 (±0.15)**	**−3.11 (0.002)**	0.03 (±0.06)	0.63 (0.53)
Stomatal conductance	**−0.97 (±0.28)**	**−3.5 (0.0004)**	−0.02 (±0.08)	−0.15 (0.88)
*Tolerance traits*				
Chlorogenic acid	0.26 (±0.16)	1.88 (0.06)	−0.02 (±0.04)	−0.92 (0.36)
Phenolic acid	−0.10 (±0.18)	−0.49 (0.62)	−0.02 (±0.04)	−0.15 (0.88)
Flavonoid	0.21 (±0.14)	0.81 (0.42)	0.04 (±0.03)	0.98 (0.33)

The *z‐* and *P*‐values were obtained using the aster model (dry treatment, df = 76) or negative binomial model (wet treatment, df = 78) with emboldened values indicating significance.

### Alignment between clinal divergence and aridity‐mediated selection

**Fig. 4:**
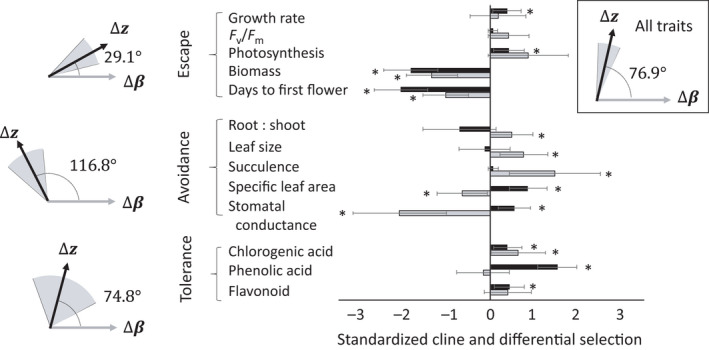
Alignment between vectors of capeweed’s clinal divergence (Δ ***z***, in black) and differential selection between wet and dry environmental treatments (Δ ***β***, in grey). Clinal divergence values (black bars) indicate slopes relative to source location aridity, and differential selection values (grey bars) indicate the differences in estimated linear selection gradients between dry and wet treatments (Δ ***β* = *β*_dry_** − ***β*_wet_**). The means of Δ ***z*** and Δ ***β*** values are presented as standardized values (zero mean, unit variance) within each group for easier contrast. Whiskers represent upper and lower 95% credible intervals (CI) of Markov chain Monte Carlo estimates, and asterisks indicate no overlap in CIs with zero. The top‐right panel shows the estimated angle between vectors of clinal divergence (Δ ***z***) and differential selection (Δ ***β***) for all 13 traits, with the grey‐shaded area representing 95% CI in the estimate of the angle. Left‐hand vectors and angles show the results for each of the three trait modules or ‘strategies’ of plant drought resistance, including traits facilitating drought escape, avoidance and tolerance.

If aridity‐mediated selection drives local adaptation for each of our 13 traits, then the vector of clinal divergence along the aridity gradient (Δ ***z***) should align with the vector of differential selection between dry and wet conditions (Δ ***β***) (Fig. S1; see the Supporting Information for detailed theoretical predictions). The vector of clinal divergence for all 13 traits was weakly aligned with the vector of differential selection (Fig. 4, upper right panel; estimated angle: *θ* = 76.9°, CI = (65.4°, 87.9°)). However, when we partitioned traits into modules of drought resistance (i.e. drought escape, avoidance and tolerance strategies), drought escape traits showed strong alignment between differential selection and clinal divergence (*θ *= 29.1°, CI = (13.8°, 46.5°); significantly less than orthogonal), and drought tolerance traits showed moderate, but nonsignificant, alignment (*θ *= 74.8°, CI = (27.5°, 110.1°)) (Fig. 4). By contrast, clinal divergence of dehydration avoidance traits was opposite to the direction of selection imposed by aridity (*θ* = 116.7°, CI = (94.6°, 138.6°)), with the angle between vectors significantly greater than orthogonal (Fig. 4). Thus, alignment between aridity‐mediated selection and clinal divergence for two trait modules is obscured, in the pooled analysis of all 13 traits, by misalignment between selection and divergence primarily in dehydration avoidance traits.

## Discussion

We hypothesized that aridity‐mediated selection on drought resistance traits contributes to local adaptation and population differentiation in invasive capeweed populations in Australia. Our results are consistent with this hypothesis, given that: (1) capeweed populations from arid regions in Australia perform better in experimentally dry conditions than populations sourced from wetter regions; (2) several traits mediating drought stress in plants have diverged across natural aridity gradients in capeweed’s invasive range; (3) such traits are subject to divergent phenotypic selection in wet vs dry environmental treatments; and (4) aridity‐mediated selection predicts multivariate orientations of clinal divergence across aridity gradients in Australia, particularly for traits involved in drought escape. Our findings suggest that conditions of aridity in nature may be important agents of selection for local adaptation, in agreement with results from observational studies of selection in the field (see Caruso *et al*., 2017, 2020; Siepielski *et al*., 2017).

### Aridity predicts divergence for drought resistance

Populations derived from arid regions in Australia had higher fitness in experimental dry treatments than populations from wetter regions, consistent with the hypothesis that capeweed populations are adapted to local aridity conditions across their invasive range. We did not, however, observe the reciprocal pattern – a significantly negative association between aridity of origin and population fitness under experimental wet conditions – that would indicate a fitness trade‐off between treatments, although our point estimate was negative, and we may simply lack the power (given our eight populations) to confirm a trade‐off. Reciprocal transplant experiments in natural habitats test for local adaptation to the full suite of environmental variables that define local conditions in nature (Kawecki & Ebert, 2004), yet they tell us far less about the specific environmental factors to which populations are locally adapted (Wade & Kalisz, 1990; Wadgymar *et al*., 2017b). Our experiments demonstrate the importance of drought stress as a potential agent of local adaptation, although we acknowledge that other abiotic and biotic environmental variables also might contribute to local adaptation in nature (Kawecki & Ebert, 2004). For instance, plants in arid environments may experience earlier declines in pollinator abundance *in situ* compared with our field experimental site, where pollinators remained abundant throughout the experiment. Earlier pollinator declines should further increase selection for early flowering in arid environments and amplify fitness trade‐offs in nature.

### Clinal divergence of drought resistance traits

Consistent with previous work in other plant systems (e.g. Manzaneda *et al*., 2015), our drought resistance traits showed plastic responses to water treatments in our experiments. Populations sampled across Australian aridity gradients also have diverged for some of these traits. Although individual traits showed varying degrees of clinal divergence (Tables S2, S3; Fig. S2), phenotypes showed consistent patterns of multivariate trait divergence in glasshouse and field experiments. Overall, Australian capeweed populations showed predictable patterns of divergence based on local aridity conditions, similar to latitudinal clines reported in other invasive plant species (Maron *et al*., 2004; Etterson *et al*., 2008; Colautti & Barrett, 2013; van Boheemen *et al*., 2019a).

Because we measured trait expression in common gardens, phenotypic differences among populations should be caused predominantly by genetic differences. Nevertheless, maternal effects also may have contributed to population differences as our experimental individuals were grown from field‐collected seeds (Rossiter, 1996). Previous drought and heat studies documented transgenerational maternal effects through seed provisioning and epigenetic modification in several species (Johannes *et al*., 2009; Herman *et al*., 2012; Groot *et al*., 2017; Tabassum *et al*., 2017), with exposure to aridity in previous generations inducing faster growth, earlier flowering, and greater survival and seed production (Herman & Sultan, 2011; Herman *et al*., 2012; Groot *et al*., 2017; Tabassum *et al*., 2017), and with greater magnitudes of induction in genotypes from arid localities (Groot *et al*., 2017). Such adaptive transgenerational effects, if present in capeweed, could inflate estimates of genetic effects on clines observed in common gardens, requiring caution when interpreting our results.

While acknowledging potential biases in estimating clines without controlling for maternal effects, we nevertheless suggest that the contributions of maternal effects to observed clinal patterns may be weaker than genetic effects for several reasons. First, although we expect adaptive maternal effects to amplify clinal patterns in dry relative to wet experimental treatments (i.e. aridity × treatment interactions; Herman *et al*., 2012; Groot *et al*., 2017; Tabassum *et al*., 2017), we found little evidence for such interactions. An interaction was observed only for seed set (our fitness measure), as predicted under scenarios of local adaptation to aridity. Second, maternal effects on seed set may be weak relative to effects of genotype. In *Arabidopsis thaliana,* maternal effects on seedling growth and flowering onset are comparable with the effects of genotype, whereas genotype effects overwhelmed maternal effects for seed size and total seed production (Groot *et al*., 2017), in alignment with earlier studies showing that maternal effects may be less likely to affect later stages of plant development (Rossiter, 1996). Third, although we cannot exclude epigenetically based maternal effects, maternal provisioning in capeweed is likely to be minimal due to small seed size (*c*. 1 mm × 2 mm), and because we observe no relationship between seed mass and expression of our 13 focal traits (Table S4). Finally, adaptive maternal effects may contribute little to the trait variation among populations when environmental variation occurs at a greater spatial scale (e.g. across the entire aridity gradient in capeweed) than the extent of geneflow (Galloway, 2005; Montague *et al*., 2008), although population genetic analyses will be required to evaluate whether this applies to invasive capeweed populations.

### Multivariate selection in wet vs dry environments

Directional selection differed substantially between wet and dry treatments in both multivariate orientation and strength. These results support the hypothesis that water stress is an important predictor of natural variation in selection, as implied by field studies (Siepielski *et al*., 2017). Our results also are consistent with the idea that selection becomes stronger in stressful environments (Caruso *et al*., 2017), possibly because stress increases displacement of populations from their optima (Kingsolver *et al*., 2001).

Local adaptation may rely on mutations or traits under opposing directional selection between environments, or those that are selectively favoured in some environments and neutral in others (i.e. conditional neutrality; Hall *et al*., 2010; Anderson *et al*., 2013). In capeweed, directional selection on biomass acted antagonistically between wet and dry treatments, with selection favouring larger size under wet conditions and smaller size under dry. However, we found that traits more often exhibited significant linear selection in only one environmental treatment: thus, directional selection on root‐to‐shoot ratio in the wet treatment (but not dry), and selection on flowering time, leaf size, leaf succulence and stomatal conductance in the dry treatment (but not wet). These results are consistent with findings from Siepielski *et al*. (2013) that spatial changes in selection were due primarily to variation in its strength rather than its direction. Studies manipulating water environments likewise found antagonistic directional selection to be rare (Heschel *et al*., 2004; Volis *et al*., 2004; Heschel & Riginos, 2005; Sherrard & Maherali, 2006; Brachi *et al*., 2012; Ivy & Carr, 2012; Kenney *et al*., 2014; Lambrecht *et al*., 2017; Hamann *et al*., 2018). Nevertheless, it is important to note that environmental differences in the strength, but not the direction, of selection in univariate trait space generates discordant multivariate directional selection (i.e. nonzero angles between selection gradient vectors), and that concordant directional selection between environments in univariate analyses *does not* imply an absence of fitness trade‐offs between environments (Connallon & Clark, 2014; Martin & Lenormand, 2015).

### Aridity‐mediated selection predicts divergence along natural aridity gradients

The nearly orthogonal orientation between the vector of differential selection between wet and dry treatments (Δ ***β***) and the vector of clinal divergence across natural aridity gradients (Δ ***z***) could have at least two causes. First, for trait sets that *are* locally adapted (i.e. each trait closely tracks its local optimum in nature), a 90° angle implies that environmental variables other than aridity underlie geographical variation in selection and divergence among populations (i.e. local aridity poorly predicts local trait optima: ρ*_bc_* = 0 in Fig. S1). Alternatively, and contrary to our model, genetic constraints may hinder local adaptation of some traits, leading to an overall mismatch between aridity‐mediated selection and clinal divergence in which some traits fail to track their local optima (Dudley, 1996a,b; Duputié *et al*., 2012). In support of this second explanation, we find that dehydration avoidance traits are maladapted with respect to drought adaptation, which accounts for the global mismatch between Δ ***β*** and Δ ***z***.

Univariate tests reveal alignment between selection and divergence for several drought escape and tolerance traits, with plants from arid sites flowering earlier, achieving lower final biomass and producing more chlorogenic acid. The imperfect multivariate alignment between selection and divergence for these drought adaptation modules may be attributable to heterogeneity among traits in the strength of stabilizing selection (see Fig. S1). Patterns of divergence for capeweed drought escape traits parallel those of other annual plants distributed across aridity gradients (Stinson, 2004; Heschel & Riginos, 2005; Sherrard & Maherali, 2006; Franks, 2011; Kenney *et al*., 2014; Wolfe & Tonsor, 2014; Kooyers *et al*., 2015). Although tests of drought‐related selection and divergence rarely focus on plant chemical traits (Berardi *et al*., 2016; Vaknin & Mogilevski, 2019; Sullivan & Koski, 2021), which more often are considered in contexts of herbivore defence (Feeny, 1970; Simmonds, 2003; but see Salehin *et al*., 2019), our results suggest that high leaf concentrations of chlorogenic acid are adaptive in arid environments, possibly because they limit oxidative damage (Grace *et al*., 1998; Close & McArthur, 2002).

Selection and clinal divergence of dehydration avoidance strategy traits were negatively correlated, with selection in the dry treatment favouring phenotypes that facilitate water retention, yet populations from arid regions expressing traits that *exacerbate* water loss. Such counter‐gradients between selection and clinal divergence could arise from physiological or developmental constrains in annual plants that experience strong selection for early phenology (Stinson, 2004; Heschel & Riginos, 2005; Sherrard & Maherali, 2006; Kenney *et al*., 2014; Wolfe & Tonsor, 2014). For example, rapid growth and early reproduction (drought escape) are accomplished by increasing photosynthetic rates through enhanced gas exchange (Ackerly *et al*., 2000) and ability to harvest light energy via increased specific leaf area, which could increase water loss (Dudley, 1996a; Geber & Dawson, 1997). In our dataset, there is a strong, positive phenotypic correlation between stomatal conductance and photosynthetic rate (Table S5a), which implies a trade‐off between drought escape and avoidance strategies that may lead to counter‐gradient clines in dehydration avoidance traits (see Geber & Dawson, 1990, 1997; Geber, 1990; Ackerly *et al*., 2000; Manzaneda *et al*., 2015; Campitelli *et al*., 2016). Likewise, although aridity did not affect selection on the unknown phenolic acid and flavonoid, both diverged among capeweed populations, perhaps as an indirect response to selection on the chlorogenic acid, with which these compounds are correlated (Table S5b).

Clinal divergence of drought escape and avoidance traits in capeweed show parallels with the trait divergence documented in a 10‐yr experimental evolution study (using *Biscutella didyma*; Metz *et al*., 2020), which reported rapid divergence in flowering phenology and allocation to reproduction, yet no divergence in water conservation traits. Their results and ours suggest that drought escape, which is driven predominantly by plant phenology, may be more evolutionarily labile than dehydration avoidance strategies.

### Conclusion

We have presented a comprehensive analysis of the effects of water stress on selection and divergence along aridity gradients of invasive capeweed populations in Australia. Our results suggest that aridity is probably a key driver of geographical divergence in drought escape and tolerance strategies of invasive capeweed. The mismatch between selection and divergence of dehydration avoidance traits could reflect trade‐offs between drought escape and avoidance strategies, although formal quantitative genetic analyses are required to confirm that genetic constraints hinder adaptation of dehydration avoidance traits. We suggest that adaptation of capeweed to drought stress may have contributed to its spread within Australia (Colautti & Barrett, 2013).

## Author contributions

AU, JLM and RMG designed the study; CC and RD collected the data; CC, RD, AU and TC contributed to analyses; AU and TC wrote the paper; and all authors contributed to editing. CC and RD contributed equally to this work.

## Supporting information


**Fig. S1** Theoretical relation between differences in multivariate directional selection between manipulated environments and patterns of clinal divergence across natural environmental gradients.
**Fig. S2** Estimates of clinal divergence in 13 focal traits along aridity gradients.
**Notes S1** Theoretical relation between differences in multivariate directional selection and patterns of clinal divergence across natural environmental gradients.
**Table S1** Sites of seed collection and source location aridity.
**Table S2** Univariate maximum‐likelihood estimates of the effect of source location aridity on trait expression.
**Table S3** Univariate Bayesian estimates of the effect of source location aridity on trait expression.
**Table S4** The relationship between average seed mass and multivariate drought resistance phenotypes.
**Table S5** Phenotypic correlations among selected traits.
Please note: Wiley Blackwell are not responsible for the content or functionality of any Supporting Information supplied by the authors. Any queries (other than missing material) should be directed to the *New Phytologist* Central Office.

## Data Availability

Data are deposited in Dryad, doi: 10.5061/dryad.tht76hf17.
