# Supplementary material for: Multivariate selection mediated by aridity predicts divergence of drought‐resistant traits along natural aridity gradients of an invasive weed
Source: New Phytol. 2022 Feb 28;234(3):1088–100. doi: 10.1111/nph.18018 (PMC9311224; doi:10.1111/nph.18018)
Supplement: Supplementary file 1 — Fig. S1 Theoretical relation between differences in multivariate directional selection between manipulated environments and patterns of clinal divergence across natural environmental gradients. Fig. S2 Estimates of clinal divergence in 13 focal traits along aridity gradients. Notes S1 Theoretical relation between differences in multivariate directional selection and patterns of clinal divergence across natural environmental gradients. Table S1 Sites of seed collection and source location aridity. Table S2 Univariate maximum‐likelihood estimates of the effect of source location aridity on trait expression. Table S3 Univariate Bayesian estimates of the effect of source location aridity on trait expression. Table S4 The relationship between average seed mass and multivariate drought resistance phenotypes. Table S5 Phenotypic correlations among selected traits. Please note: Wiley Blackwell are not responsible for the content or functionality of any Supporting Information supplied by the authors. Any queries (other than missing material) should be directed to the New Phytologist Central Office. [file NPH-234-1088-s001.pdf]

New Phytologist

New Phytologist Supporting Information

Article title: Multivariate selection mediated by aridity predicts divergence of drought resistant traits along natural aridity gradients of an invasive weed

Authors: Carter Carvalho, Rochelle Davis, Tim Connallon, Roslyn M. Gleadow, Joslin L. Moore, Akane Uesugi

Article acceptance date: 26 January 2022

The following Supporting Information is available for this article:

Fig. S1 Theoretical relation between differences in multivariate directional selection between manipulated environments and patterns of clinal divergence across natural environmental gradients.

Fig. S2 Estimates of clinal divergence in 13 focal traits along aridity gradients.

Table S1 Sites of seed collection and source location aridity.

Table S2 Univariate maximum likelihood estimates of the effect of source location aridity on trait expression.

Table S3 Univariate Bayesian estimates of the effect of source location aridity on trait expression.

Table S4 The relationship between average seed mass and multivariate drought resistance phenotypes.

Table S5 Phenotypic correlations among selected traits.

Notes S1 Theoretical relation between differences in multivariate directional selection and patterns of clinal divergence across natural environmental gradients.

Fig. S1 Theoretical relation between differences in multivariate directional selection between manipulated environments ($\Delta\boldsymbol{\beta}$: the vector of differences in selection between a pair of environmental treatments, *e.g.*: wet versus dry) and patterns of clinal divergence across natural environmental gradients ($\Delta\boldsymbol{z}$: the vector of trait cline slopes). The model assumes that phenotypic optima for *n* genetically uncorrelated quantitative traits shift linearly across a natural environmental gradient in the species' range with local adaptation causing trait clines to track local trait optima ($\Delta\boldsymbol{z} = \boldsymbol{c}$, where $\boldsymbol{c} = \{c_1, c_2, \ldots c_n\}$ describes the slopes of the *n* trait optima across the environmental gradient). Changes in a manipulated environmental variable (*e.g.*, aridity) cause the *n* trait optima to shift linearly with slopes $\boldsymbol{b} = \{b_1, b_2, \ldots b_n\}$; ρ_{bc} (the cosine similarity between vectors $\boldsymbol{b}$ and $\boldsymbol{c}$, which is conceptually similar to a correlation coefficient, having a range between -1 and 1) captures the degree to which changes in the manipulated environmental variable predict clinal divergence across the natural gradient. Three examples are shown in which: (**A**) cline slopes are negatively correlated with optimum shifts associated with the manipulated environmental variable (ρ_{bc} = -3/4, corresponding to an angle between $\Delta\boldsymbol{\beta}$ and $\Delta\boldsymbol{z}$ in excess of 90°); (**B**) clines lopes are uncorrelated with optimum shifts across manipulated environments (ρ_{bc} = 0, corresponding to an angle of 90°); and (C) there is strong alignment between vectors $\Delta\boldsymbol{\beta}$ and $\Delta\boldsymbol{z}$ (*i.e.*, a small angle between them), which implies that natural variation in the manipulated environmental variable predicts the direction of clinal divergence (*i.e.*, angles less than 90° imply that $\rho_{bc} > 0$, as in example **C** at the bottom right). Variation among traits in the strength of stabilizing selection (represented by *S*, which has a mean and variance among traits of $\bar{S}$ and var(*S*), respectively) weakens the relation between ρ_{bc} and the angle between vectors $\Delta\boldsymbol{\beta}$ and $\Delta\boldsymbol{z}$. Details of the model are presented in the Notes **S1**.

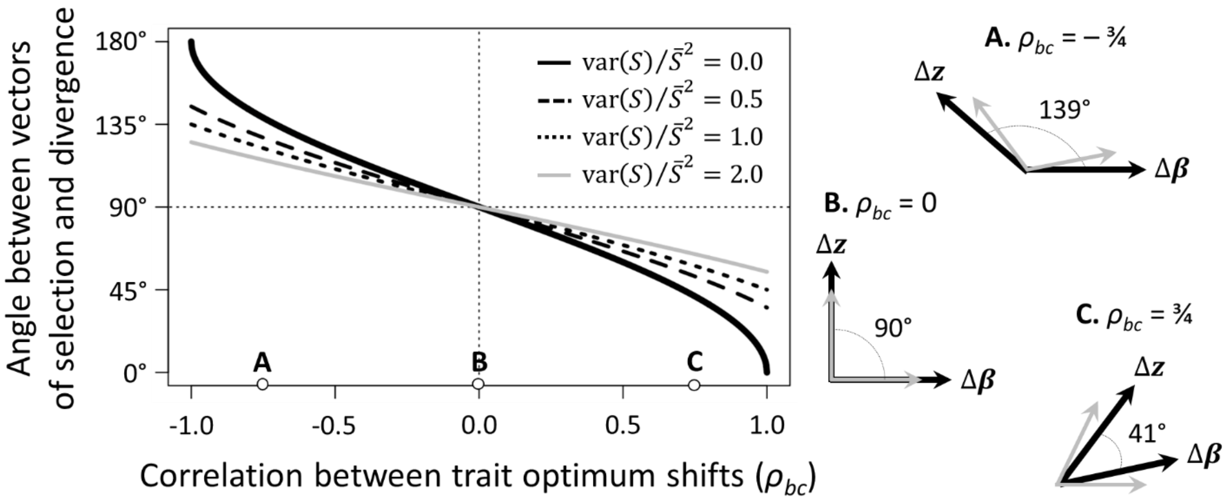

Angle between vectors of selection and divergence
180°
135°
90°
45°
0°
var(S)/S̄² = 0.0
var(S)/S̄² = 0.5
var(S)/S̄² = 1.0
var(S)/S̄² = 2.0
A
B
C
-1.0
-0.5
0.0
0.5
1.0
Correlation between trait optimum shifts (ρbc)
A. ρbc = – ¾
Δz
139°
Δβ
B. ρbc = 0
Δz
90°
Δβ
C. ρbc = ¾
Δz
41°
Δβ

Fig. S2 Estimates of clinal divergence in 13 focal traits along aridity gradients. Cline slopes were estimated, using a Bayesian framework, for each combination of experiment and water treatment. Error bars indicate 95% CI. Asterisks indicate non-overlap of CIs with zero. † Many samples (99 out of 120) in the dry treatment of the 2018 greenhouse experiment failed to flower and, thus, the estimated slope for this trait was not reliable for that particular experiment and treatment.

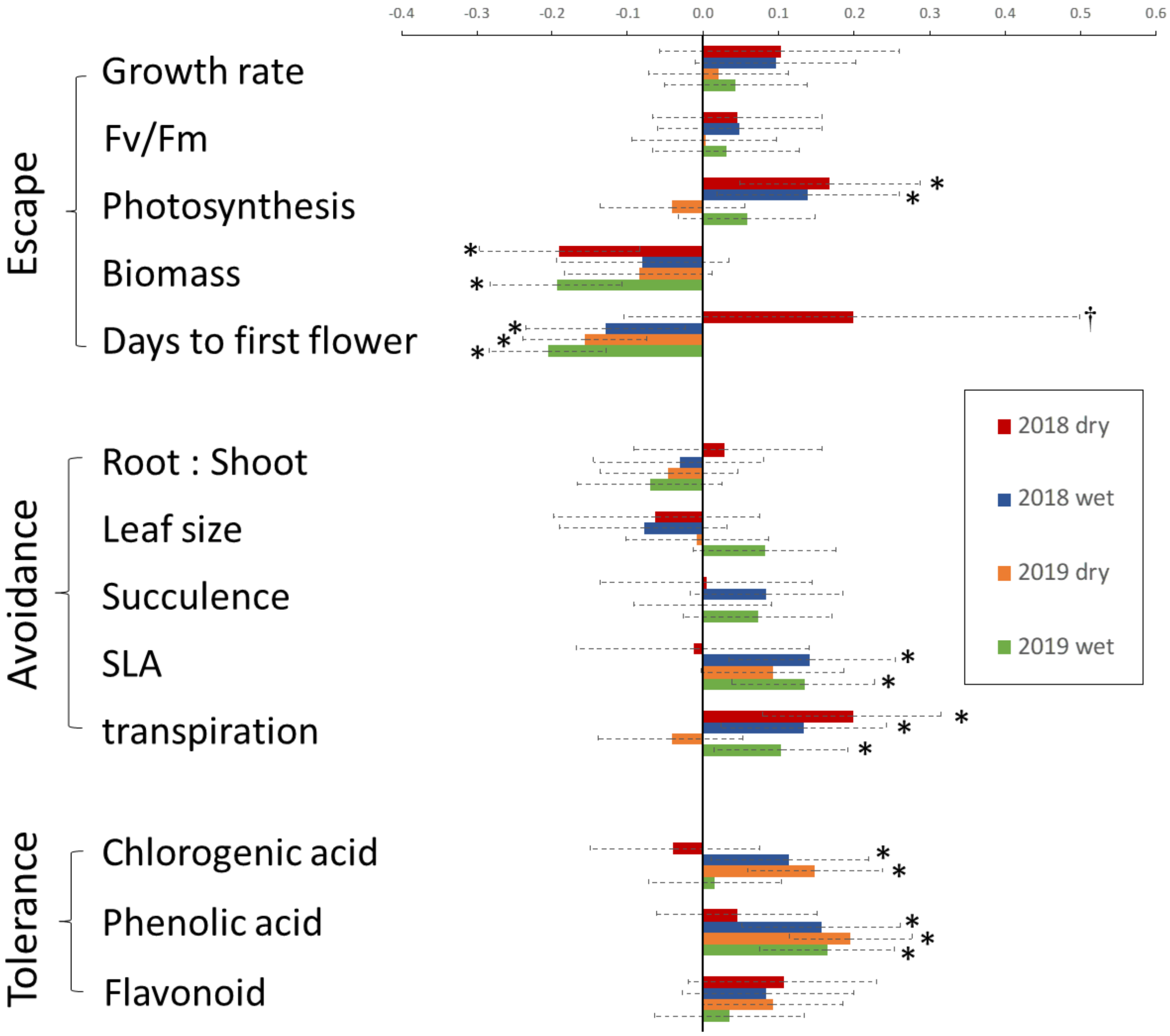

Table S1 Sites of seed collection and source location aridity. Transect regions: EAU = eastern Australia, WAU = western Australia. N indicates sample size used in 2018 (greenhouse) and 2019 (field) experiments. Numbers in parenthesis (2018 greenhouse experiment) indicate number of capeweed patches from which seeds were collected from.

Population	Transect region	Latitude	Longitude	Aridity index	N (2018)	N (2019)
Mornington	EAU	-38.461	144.905	1.55	48 (6)	35
Modewarre	EAU	-38.266	144.122	2.13		21
Bentleigh	EAU	-37.921	145.067	2.00	48 (6)	
Bendigo	EAU	-36.757	144.279	2.70	46 (5)	
Charlton	EAU	-36.268	143.351	4.34	46 (6)	25
Sea Lake	EAU	-35.506	142.853	5.83	34 (5)	33
Mildura	EAU	-34.208	142.125	6.82	19 (3)	
Sydney	EAU	-33.869	151.209	1.27		10
Ridgefield	WAU	-32.494	116.986	4.42		25
Perth	WAU	-31.951	115.860	2.69		7
Perenjori	WAU	-29.465	116.485	8.06		34

Table S2 Univariate maximum likelihood estimates (using the emtrends function) of the effect of source location aridity on trait expression in the 2018 greenhouse and 2019 field experiments, each carried out under dry and wet treatments. The mean estimates of slope and the lower and upper 95% confident intervals (CI, in { }) are presented. Values in bold indicate non-overlap of CIs with zero.

	Dry treatment	Wet treatment
**2018 Greenhouse**		
Growth rate	0.008 {-0.003, 0.02}	**0.012 {0.001, 0.024}**
Fv/Fm	-0.001 {-0.002, 0.001}	0 {-0.002, 0.002}
Photosynthesis	**0.45 {0.06, 0.84}**	**0.43 {0.035, 0.82}**
Biomass	**-0.045 {-0.081, -0.009}**	-0.036 {-0.072, 0.001}
Days to flowering	0.151 {-0.067, 0.37}	-0.092 {-0.25, 0.069}
Root-to-shoot ratio	0.005 {-0.026, 0.036}	-0.003 {-0.034, 0.028}
Leaf size	-0.025 {-0.081, 0.032}	-0.037 {-0.094, 0.02}
Succulence	-0.008 {-0.16, 0.15}	0.13 {-0.026, 0.29}
SLA	-0.004 {-0.022, 0.014}	**0.039 {0.021, 0.057}**
Stomatal conductance	**0.011 {0.001, 0.021}**	**0.012 {0.002, 0.023}**
Chlorogenic acid	-0.02 {-0.088, 0.048}	0.051 {-0.018, 0.12}
Unknown phenolic acid	0.12 {-0.39, 0.63}	**0.55 {0.035, 1.06}**
Unknown flavonoid	**0.12 {0.036, 0.21}**	**0.11 {0.022, 0.20}**
**2019 Field**		
Growth rate	0.001 {-0.015, 0.018}	0.004 {-0.013, 0.021}
Fv/Fm	0 {-0.003, 0.003}	0.001 {-0.002, 0.003}
Photosynthesis	-0.182 {-0.74, 0.38}	0.243 {-0.32, 0.80}
Biomass	**-0.045 {-0.089, -0.002}**	**-0.079 {-0.12, -0.035}**
Days to flowering	-0.038 {-0.077, 0.001}	**-0.056 {-0.095, -0.017}**
Root-to-shoot ratio	-0.042 {-0.12, 0.034}	-0.038 {-0.11, 0.039}
Leaf size	-0.005 {-0.051, 0.04}	0.024 {-0.021, 0.07}
Succulence	-0.008 {-0.17, 0.15}	0.091 {-0.067, 0.25}
SLA	0.036 {-0.015, 0.087}	0.041 {-0.01, 0.092}
Stomatal conductance	-0.003 {-0.025, 0.019}	0.015 {-0.007, 0.037}
Chlorogenic acid	0.036 {-0.008, 0.079}	0.003 {-0.041, 0.046}
Unknown phenolic acid	**0.29 {0.12, 0.45}**	**0.27 {0.10, 0.43}**
Unknown flavonoid	0.097 {-0.021, 0.21}	0.047 {-0.071, 0.16}

Table S3 Univariate Bayesian estimates of the effect of source location aridity on trait expression in the 2018 greenhouse and 2019 field experiments. The mean estimates of cline slope and the lower and upper 95% credible intervals (CI) are presented. Values in bold indicate non-overlap of CIs with zero.

	Estimate	Lower CI	Upper CI
Growth rate	**0.055**	**0.009**	**0.101**
Fv/Fm	0.027	-0.020	0.074
Photosynthesis	**0.059**	**0.011**	**0.107**
Biomass	**-0.138**	**-0.184**	**-0.091**
Days to flowering	**-0.158**	**-0.205**	**-0.111**
Root-to-shoot ratio	-0.040	-0.087	0.008
Leaf size	0.010	-0.037	0.059
Succulence	0.028	-0.021	0.077
SLA	**0.097**	**0.048**	**0.147**
Stomatal conductance	**0.070**	**0.023**	**0.117**
Chlorogenic acid	**0.056**	**0.010**	**0.103**
Unknown phenolic acid	**0.156**	**0.110**	**0.202**
Unknown flavonoid	**0.060**	**0.013**	**0.107**

Table S4 Post hoc MANCOVA results examining the relationship between average seed mass and multivariate drought resistance phenotypes in the 2019 field experiment. Because we did not weigh seeds that we used in the experiment, we approximated the seed size of each experimental plant by obtaining the mean seed mass of its maternal family (assuming that maternal imput is similar among sibs). We took the average of five seeds per maternal family. A MANCOVA was conducted for 13 traits as response variables and the mean seed mass, treatment, and their interactions as predictor variables.

	Pillai's index	$F_{13,118}$	*P*
Seed mass	0.10	1.02	0.43
Treatment	0.84	46.9	< 0.0001
Seed mass x Treatment	0.15	1.63	0.084

Table S5 Post hoc analyses of phenotypic correlations among selected traits. We tested two hypotheses: a) that drought escape traits (represented by growth and photosynthetic rates) and dehydration avoidance traits (represented by stomatal conductance and specific leaf area, SLA) are phenotypically correlated, and b) that phenolic compounds (chlorogenic acid, unknown phenolic acid and flavonoid) are correlated (e.g., due to their shared biosynthetic pathways). For each set of traits, we analysed trait correlations with multivariate mixed models (MCMCglmm) with treatment and experiment as fixed effects, and population as a random effect. Values under the diagonal indicate the estimated correlation coefficients, with values in bold indicate that 95% credible intervals (CI) for estimates (values above diagonal) do no overlap with zero.

a) Phenotypic correlations among drought escape & avoidance

	Growth	Photosynthesis	Stomatal conductance	SLA
Growth	--	{-0.13, 0.06}	{-0.10, 0.08}	{-0.045, 0.12}
Photosynthesis	-0.035	--	{0.71, 0.93}	{-0.02, 0.15}
Stomatal conductance	-0.011	**0.82**	--	{0.007, 0.18}
SLA	0.36	0.06	**0.09**	--

b) Phenotypic correlations among phenolic compounds

	Chlorogenic acid	Phenolic acid	Flavonoid
Chlorogenic acid	--	{0.34, 0.50}	{0.22, 0.38}
Phenolic acid	**0.42**	--	{0.27, 0.44}
Flavonoid	**0.30**	**0.35**	--

Notes S1 Theoretical relation between differences in multivariate directional selection and patterns of clinal divergence across natural environmental gradients.

Consider a simple model of *n* quantitative traits that have evolutionarily diverged across the species' range in response to local selection. In the following calculations, we ignore effects of phenotypic plasticity on selection and trait divergence. Inclusion of plasticity should not greatly affect our predictions, as plastic responses will simply alter the *effective* amounts of change of the trait optima in the model below.

Predictions from our model apply to cases in which local adaptation is unconstrained by:

- demographic factors, *i.e.*: we assume that maladaptive gene flow and genetic drift are sufficiently weak so that local adaptation dominates.
- genetic or developmental correlations among traits, *i.e.*: we assume that the focal traits are sufficiently uncorrelated that they can each evolutionarily respond to local selection.

As we discuss in the main text, the presence of either class of constraint should tend to eliminate local adaptation, and thereby cause mismatches between the patterns of selection we observe in experimentally manipulated environments (*e.g.*, selection in wet versus dry environments) and patterns of clinal divergence across the natural environmental gradients.

Clines in trait divergence across a natural environmental gradient

We assume that each population within the species' range has locally adapted so that the mean expression of each of their traits matches its local optimum. As already mentioned, the assumption should be reasonable provided: (1) local selection is strong relative to gene flow across the range (García-Ramos & Kirkpatrick, 1997; Kirkpatrick & Barton, 1997), and (2) traits are sufficiently uncorrelated (genetically) that they can evolutionarily respond to local selection, independent of selection on other traits (see Duputié et al., 2012).

We further assume that optimum trait values change linearly across natural environmental gradients in the species' range, in which case the mean expression value for the *i*th trait of individuals in environment *x* within the natural gradient will be:

$$\bar{z}_i(x) = a_i + c_i x$$

where c_i is the slope of the trait cline across the natural gradient and a_i represents the mean trait value in a reference environment. The vector of cline slopes for the set of traits (*i.e.*, slopes of regressions of trait means on the environmental variable, x) is given by:

$$\Delta\boldsymbol{z} = [c_1, c_2, \ldots, c_{n-1}, c_n]$$

Selection gradients across experimentally manipulated environments

Now consider an experimental population in which selection is estimated in each of a pair of experimentally manipulated common garden treatments (*e.g.*, a dry and a wet treatment). We assume that, in either environment, the *i*th trait follows a distribution with mean of $\bar{z}_i$ and unit variance. We also assume that fitness is a Gaussian function of multivariate trait expression, with trait optima changing linearly with changes in the experimentally manipulated environmental variable, and other aspects of the fitness function remaining constant. Following standard quantitative genetics theory, the linear selection gradient for the *i*th trait in the *j*th environment will be:

$$\beta_{i,j} = S_i(O_{i,j} - \bar{z}_i)$$

where S_i is the strength of stabilizing selection on the *i*th trait, and $O_{i,j}$ is the trait's optimum in environment j.

Because trait optima change linearly with the manipulated environmental variable, we can define trait optima within a pair of experimental environments (treatment "A" and treatment "B") as $O_{i,A} = O_i + \varepsilon_A b_i$ and $O_{i,B} = O_i + \varepsilon_B b_i$, where ε_A and ε_B represent the state of the environmental variable in the two treatments, O_i represents the optimum in a reference environment, and b_i is the slope of the change in the trait optimum with changes in the manipulated environmental variable. The difference in the *i*th selection gradient between environments *A* and *B* will be:

$$\Delta\beta_i = \beta_{i,A} - \beta_{i,B} = S_i b_i(\varepsilon_A - \varepsilon_B)$$

The vector of differential selection between the pair of environments is:

$$\Delta\boldsymbol{\beta} = [S_1 b_1(\varepsilon_A - \varepsilon_B), S_2 b_2(\varepsilon_A - \varepsilon_B), \ldots, S_{n-1} b_{n-1}(\varepsilon_A - \varepsilon_B), S_n b_n(\varepsilon_A - \varepsilon_B)]$$

The angle between $\Delta\boldsymbol{z}$ and $\Delta\boldsymbol{\beta}$

Letting A represent the more extreme of the two environments (*e.g.*, if the environmental variable is aridity, then *A* is relatively dry and *B* is relatively wet), then the angle between the vector of selection gradient differences between environments *A* and *B* and the vector of cline slopes across a natural gradient of the same environmental variable will be:

$$\theta = \cos^{-1}\left(\frac{\Delta\boldsymbol{\beta}\cdot\Delta\boldsymbol{z}}{\|\Delta\boldsymbol{\beta}\|\|\Delta\boldsymbol{z}\|}\right) = \cos^{-1}\left(\frac{\sum_{i=1}^{n} S_i b_i c_i}{\sqrt{(\sum_{i=1}^{n} S_i^2 b_i^2)(\sum_{i=1}^{n} c_i^2)}}\right)$$

Assuming that the sensitivities of trait optima to changes in the environment are uncorrelated with the strengths of stabilizing selection on the set of traits (*i.e.*, S_i varies independently of b_i and c_i), then the angle simplifies to:

$$\theta = \cos^{-1}\left(\frac{\bar{S}\sum_{i=1}^{n} b_i c_i}{\sqrt{\overline{S^2}(\sum_{i=1}^{n} b_i^2)(\sum_{i=1}^{n} c_i^2)}}\right) = \cos^{-1}\left(\frac{\rho_{bc}}{\sqrt{1+\mathrm{var}(S)/\bar{S}^2}}\right) \quad \text{(S1),}$$

where $\bar{S}$ and var(S) represent the mean and variance of the strength of stabilizing selection across traits, $\overline{S^2} = \mathrm{var}(S) + \bar{S}^2$, and ρ_{bc} is the cosine similarity between vectors trait optimum shifts across experimental environments and trait optimum shifts along the natural environmental gradient, *i.e.*:

$$\rho_{bc} = \frac{\boldsymbol{b} \cdot \Delta\boldsymbol{z}}{\|\boldsymbol{b}\|\|\Delta\boldsymbol{z}\|} = \frac{\sum_{i=1}^{n} b_i c_i}{\sqrt{(\sum_{i=1}^{n} b_i^2)(\sum_{i=1}^{n} c_i^2)}}$$

The cosine similarity provides a measure of alignment between the divergence of trait optima across the natural environmental gradient and the divergence of trait optima across the manipulated environmental treatments in the common garden experiment, with $\rho_{bc} = 1$ representing a perfect positive alignment between experimental and natural environmental gradients, $\rho_{bc} = -1$ representing a perfect negative alignment between gradients, and $\rho_{bc} = 0$ representing a case of zero predictability.

Our predictions in the main text (see Fig. 2) are based on equation (S1), which assumes independence between S_i from the other variables, and there is no biological reason to expect otherwise. Nevertheless, positive correlations between S_i and b_i should tend reduce θ when $\rho_{bc} > 0$ and increase θ when $\rho_{bc} < 0$; negative correlations between S_i and b_i should do the opposite.

SUPPLEMENTARY REFERENCES

García-Ramos G, Kirkpatrick M. 1997. Genetic models of adaptation and gene flow in peripheral populations. *Evolution* **51**:21-28.

Kirkpatrick M, Barton NH. 1997. Evolution of a species' range. *The American Naturalist* 150:1-23.
